# Chronic low back pain among French healthcare workers and prognostic factors of return to work (RTW): a non-randomized controlled trial

**DOI:** 10.1186/s12995-015-0082-5

**Published:** 2015-10-29

**Authors:** B. Cougot, A. Petit, C. Paget, C. Roedlich, G. Fleury-Bahi, M. Fouquet, P. Menu, C. Dubois, C. Geraut, Y. Roquelaure, D. Tripodi

**Affiliations:** External Consultation on Occupational and Environmental Health, Department of Occupational and Environmental Health, Nantes University Hospital, 5 rue du Doyen Boquien, Nantes, F 44 093 France; Laboratory of Psychology of Pays de la Loire (LPPL - UPRES EA 4638), Nantes University - Faculty of Psychology, Nantes, F 44 312 France; Laboratory of Ergonomics & Epidemiology in Occupational Health, LEEST, UA-InVS - IFR 132, UPRES EA 4336 Faculty of Medicine, University of Angers, Angers, F 49 000 France; Physical Medicine Rehabilitation Department, Nantes University Hospital, 85 rue Saint Jacques, Nantes, F 44 093 France

## Abstract

**Background:**

Many factors influence the return to work of workers with chronic low back pain (CLBP). They have been said to vary according to socio-professional group. This study first aimed to compare prognostic factors influencing the return to work of CLBP healthcare workers (HCWs) and other workers (non-HCWs) after rehabilitation coupled with an occupational intervention. The second objective was to improve the evolution of indicators such as clinical examination, psychosocial impact and pain impact.

**Methods:**

Between 2007 and 2012, a cohort of 217 CLBP workers (54.8 %-women; mean age = 41.3 ± 9.5 years, 118 non-HCWs; 99 HCWs mainly from the public sector) was included in an ambulatory rehabilitation program (standard physiotherapy or intensive network physiotherapy) coupled with an occupational intervention. Workers completed a questionnaire and had a clinical examination at baseline and after 24 months’ follow up. Physical, social and occupational data was collected at the same time. Statistical analyses were performed to evaluate prognostic factors for return to work and compare the two worker populations.

**Results:**

There was no difference between groups for the rate of OP (occupational physician) intervention or type of physiotherapy. 77.3 % of workers returned to work after 2 years following inclusion. To be an HCW (OR 0.1; 95 % CI [0.03–0.34]), to have less than 112 sick- leave days (OR 1.00; 95 % CI [0.93–1.00]), a small fingertip-floor distance (OR 0.96; 95 % CI [0.93–0.99]), a low anxiety/depression score (OR 0.97; 95 % CI [0.95–1.00]), a low impact of CLBP on daily life (OR 0.96; 95 % CI [0.93–1.00]), and on quality of life (OR 0.98; 95 % CI [0.95–1.00]) at baseline were statistically associated with return to work after 2 years of follow up. Only the profession (workplace) was statistically associated with return to work after 2 years of follow up using multivariate analysis.

**Conclusion:**

To our knowledge, this is the first cohort study concerning predictive factors of RTW among CLBP workers after 2 years of follow up. Interventions in the work environment did not seem to predict job retention significantly. But only 50 % of the employees in both groups (HCW and non-HCW) had one intervention at their workplace after 2 years. This study underlined the fact that the type of physiotherapy with a well-trained physiotherapist used to take care of CLBP could not impact on the RTW forecast. To develop these initial results, it might be interesting to study the comparison between private and public sectors and to randomize the physiotherapeutic intervention.

## Background

Chronic low back pain (CLBP) and identification of risk factors in evolution toward chronicity has been the subject of numerous controversial works [1]. This is a chronic pain syndrome (recurrent or continuous) in the lower back region, lasting for at least 3 months (with or without radiculalgia). In the USA, the prevalence of LBP is from 15 to 45 % according to cross-sectional studies. Data from Western countries is similar. UK estimates place LBP as the biggest single cause of absence from work in 1988–89, when it was responsible for about 12.5 % of all days of sickness absence [1]. The “low back pain triangle” (organic factor, socioprofessional factor and psychological factor) makes the need for multidisciplinary care obvious. Recent studies show: the need for networked care for the patient, including health actors as well as interlocutors within the company, to enhance diagnosis and care quality [2]; the effectiveness at 6 months of 3 weeks’ multidisciplinary care on pain repercussions, disability and quality of life [3]; the slightly better impact of a functional restoration programme than of individual physiotherapy on absenteeism [4]; and the importance of educational and informational components in physical care (physical training) to improve patients’ self confidence [5].

It is important to note that the feasibility of the care content needs scientific backing for better efficiency [6]. Moreover, it seems that a rigorous theoretical approach along with patient-centered listening would make it possible to remove obstacles linked to professional constraints [7]. In France, in 2008, the HAS Haute, Autorité de Santé (French National Authority for Health), recommended the following modalities for chronic pain care: ambulatory outpatient identification, assessment, and treatment setting, or a request for an opinion from specialized structures and guidance to the attending physician. We note that the occupational physician is not included in these recommendations (Circulaire DGOS/DH n°98–47, 12). To date, few studies have investigated the factors of continued employment in the public sector, including care services. Therefore, those studies pay little attention to organizational context or intervention in the workplace before the decision to return to work.

## Aims of the study

The aim of this study was first to investigate prognosis factors for return to work at 2 years of a population of CLBP employees after care in a specialized occupational pathology consultancy with two main areas of intervention: conventional care in ambulatory functional rehabilitation and intervention by the OP in the company, comparing a population of healthcare workers (HCW) and a population of non-healthcare workers (non-HCW control group). The secondary objective was to analyze the overall effectiveness of the programme and the evolution of indicators such as clinical examination, psychosocial and pain impact.

## Methods study design

A prospective cohort study, monitoring changes for a period of two years. Potential predictors for staying at work were initially recorded at baseline (T1), and their evolution after a physiotherapeutic and occupational approach was recorded at each follow-up visit, that is at 6 months, one year and two years.

### Population

The workforce followed up by OPs in the department of Loire Atlantique (West of France) consisted of 472,483 employees in 2009. An information letter on the establishment of a Specialized Professional Pathology Consultancy for management of CLBP, based on the biopsychosocial model, along with the establishment of a link with the occupational physician or the company during sick-leave was sent out in 2006. The letter to General Practitioners (GPs) (*n* = 800) and OP (*n* = 120), explained the rules for inclusion in the programme. The initial multidisciplinary consultation was carried out by an OP, a rehabilitation physician and an occupational psychologist. This first meeting was to explore the factors involved in the LBP issue in a multidimensional perspective. If indications were raised, the patient was included in the protocol. Inclusion criteria were: to be between 18 and 65 years old, to be an employee in the care sector or outside the care sector, and to have suffered from LBP for more than three months. Exclusion criteria were: surgical indications, inflammatory disease (rheumatoid arthritis, ankylosing spondylitis…), or scalable lower back sciatica, fibromyalgia, being unemployed or self-employed.

Under a protocol of exploratory research, consultation was open to care staff in the public sector for 2 years (HCW reference group), and then to the private industrial and commercial sectors for 2 years (control group).

The intervention approach comprised two dimensions:

1. During the visit to decide on inclusion (T1) and after clinical examination by the rehabilitation physician and the OP, the principle of ambulatory physiotherapy was outlined to the patient: it was either a standard rehabilitation program with the patient’s usual physiotherapist, or a more intensive program with a physiotherapist of the Regional CLBP network. The choice was determined by the distance between home and the physiotherapist’s practice. A typical prescription was issued to the patient at the end of the consultation, explaining the principle and requesting a signed consent. Care by the patient’s usual physiotherapist (MKH) comprised three sessions of 30 min per week for 5 weeks. Care by the network physiotherapist included three one-hour sessions per week for 5 weeks. It was an active treatment excluding the use of any kind of passive technique, massage or acupuncture. The exercises were intended to promote flexibility, training, stretching, muscular strengthening, endurance working and proprioception exercises. The program also included an educational component, with learning exercises to be performed only at home. To minimize the risks associated with the resumption of sustained physical activity in the deconditioned subject, attention focused on the progressive nature of the program and on adapting techniques to the person. In addition to the 15 initial sessions, the patient might also receive five additional booster sessions during the following year.
2. During the inclusion visit (T1), a review of the curriculum and the link between pain and work was recorded by the OP and the occupational psychologist. This involved noting the patient’s speech, his feelings in specific work situations, and the constraints perceived in the description of his work. The psychic balance involved in work is connected with the meaning of work, investment of personality in work, identity investment materialized in gesture. What, in the encounter between the organization of work and investment of the subject, could develop or worsen the subject’s health?

This was a specific and single qualitative analysis of the situation. It included identifying (− if this had not already been done during the clinical interview with the doctor) satisfaction at work, historical items relating to the company and to the professional background, the quality of relationships with superiors and colleagues, pathogenic organizational practices, organizational changes, the arrival of a new manager, and new working techniques.

The various biomechanical and psychosocial risk factors were explained, as well as the interest in linking GP, OP and employer from a staying-at-work perspective. This second session took 60 min per patient on average. At the end of the consultation, a summary and a letter were dictated to the patient and sent to the principal correspondents, that is OPs and other doctors looking after the case, such as rheumatologists, physiotherapists, surgeons, pain specialists etc. The patient was sent a copy. At the end of the letter, appointments were proposed at six months (T2), twelve months (T3) and 24 months (T4). There was no interim relaunch. Follow-up consultations were carried out by the OP, who recorded the follow-up indicators.

### Follow-up

Variables recorded during the medical visit for inclusion were: the number of days’ sick leave (declarative data) before inclusion, clinical examination data, i.e. history of back pain or sciatica, smoking, comorbidity (psychiatric illness, chronic disease), level of pain intensity (visual analogic scale), weight, height, Schober index (10-cm Schober test), fingertip-floor distance, evaluation of the psychological impact of pain on social and professional life: Hamilton Anxiety Depression (HAD, Fear Avoidance Belief Questionnaire (FABQ), Dallas, and Quebec scales [8–11]. Work questionnaire: job description, employee’s satisfaction at the workplace, vision of his future career, development training, and ways to improve on his job.

Variables collected during the follow-up were: employee satisfaction at the workplace, the job maintenance of the employee at the workplace or at an adapted one, termination, sick leave absence, clinical examination data: level of pain intensity (visual analogic pain scale), weight, height, Schober index (10-cm Schober test), fingertip-floor distance, evaluation of the psychological impact of pain on social and professional life (HAD, FABQ), Dallas, and Quebec questionnaires. The work questionnaire mentioned the intervention of the OP in the subject’s company, the way toward a return to the work, the individual’s desire to keep his job or desire for a change of career.

Confronted with a large number of lost follow-up participants at two years, a recovery program was implemented from September to December 2012. A systematic callback of lost follow-up patients was organized by means of a standardized guided interview and operating engaging techniques. Patients were offered a consultation and/or the opportunity to respond to a questionnaire or to answer a few telephone questions. The primary objective here was to collect “staying at work” outcomes. The secondary objective was to improve the evolution of other indicators (clinical, psychosocial and impact of pain). The local Ethic Committee (Groupe Nantais d’Etudes Cliniques) approved the study.

### Statistical analysis

Descriptive raw data of our two “HCW” and “control” groups are presented. Comparisons of means by student test or Wilcoxon test well modeled by a normal distribution were performed, and comparisons of percentages by chi-2 or Fisher test. A 5 % degree of significance was found. Univariate and multivariate regression models were used to evaluate prognostic factors for return to work. SAS (R) software Version 9.3 was used for data analysis.

## Results

**Fig. 1:**
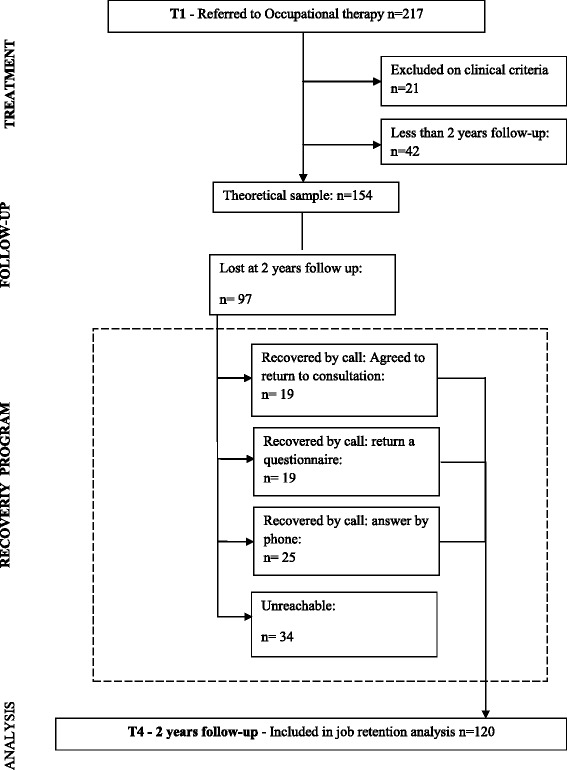
Patients flow chart through the study

Description of the sample: 217 patients were included between January 2007 and December 2012. Among them, 21 were excluded on the basis of clinical criteria and 42 because they had less than 2 years’ follow up. Consequently, 154 patients were included between January 2007 and December 2011, allowing a 2-year follow-up period. Confronted with a large number (*n* = 97) of lost follow-up participants with two years’ follow-up, a recovery program was implemented from September to December 2012. We were unable to contact 34 of the subjects at 2 years, so a total of 120 patients were included in the statistical analysis with complete data (Fig. 1). Both the two initial groups and the final group were comparable for each descriptive variable (no statistical difference due to lost follow-up patients).

Our initial cohort thus included a group of 99 carers mainly from the public sector (2/3 of them nursing assistants, 1/3 nurses and logistics services workers) and a group of 118 non-carers from the industrial or commercial sector and the private sector (drivers of heavy construction machinery, agricultural machinery drivers, construction workers and public works masons, painters, plumbers, electricians, cable installers of telecommunications agents, commercial agents’ vehicle drivers, delivery drivers, mechanics, janitors, industrial cleaning agents).

Sociodemographic data: the mean age of our population was 41.3 +/− 9.5 years with no significant difference between the two groups; there was a total of 55 % women but the carer group included 88 % women. For other variables such as: marital and social status, there was no significant difference between the two groups (Table 1).

**Table 1:** Individual patient characteristics (T1) and “caring” procedure

Individual patient characteristics	Non-HCW	HCW	Total	*p*-value
	*N* = 118	*N* = 99	*N* = 217	
Sociodemographic factors				
Age [y, mean (SD)]	42.03 (9.32)	40.49 (9.72)	41,33 (9.51)	0.2361
Age (classes) (%)				
20–29	11.02 %	18.18 %	14.29 %	/
30–39	27.12 %	25.25 %	26.27 %	
40–49	41.53 %	37.37 %	39.63 %	
50–59	19.49 %	18.18 %	18.89 %	
60–69	0.85 %	1.01 %	0.92 %	
Sex (%)				
Female	27.12 %	87.88 %	54.84 %	*< 0.001*
Male	72.88 %	12.12 %	45.16 %	
Conjugal relationship with or without child (%)				
Yes	48.18 %	40.91 %	44.95 %	0.3067
No	51.82 %	59.09 %	55.05 %	
Socio-occupational factors				
Sick leave before inclusion on the study [days, mean (SD)]	174.13 (185)	93.45 (132)	136.93(168)	*0.0002*
Work Satisfaction (%)				
Yes	74.44 %	82.89 %	78.31 %	0.1881
No	25.56 %	17.11 %	21.69 %	
Clinical factors				
BMI [kg/m^2^, mean (SD)]	24.67 (4.58)	24.04 (4.62)	24.39 (4.60)	0.3365
Weight [kg, mean (SD)]	73,95 (15.90)	66,61 (13.64)	70,63 (15.33)	*0.0005*
Height [cm, mean (SD)]	172.48 (9.53)	166.30 (8.40)	169.75 (9.54)	*<0.0001*
Cigarette Smoking [packs years, mean (SD)]	5.91 (9.70)	1.86 (4.54)	4.06 (8.01)	*0.0009*
Visual Analogic Pain Scale VAPS [mean (SD)]	4.53 (2.04)	3.33 (2.35)	3.99 (2.26)	*0.0001*
Duration of Pain [months, mean (SD)]	91.83 (93.04)	71.92 (82.66)	82.80 (88.84)	0.1011
Finger-Tip Score [cm, mean (SD)]	20.45 (14.73)	15.73 (17.40)	18.31 (16.13)	*0.0054*
Schober Index [cm, mean (SD)]	3.23 (1.42)	3.24 (1.15)	3.23 (1.30)	0.6855
Comorbidity (%)				
No-one	47.66(%)	60.00(%)	53.13(%)	0.1756
Others (cardiovascular, musculoskeletal…)	32.71(%)	28.24(%)	30.73(%)	
Phsychiatric Disorders	19.63(%)	11.76(%)	16.15(%)	
History of lumbago (%)				
Yes	53.10 %	56.38 %	54.59 %	0.6364
No	46.90 %	43.62 %	45.41 %	
History of sciatica (%)				
Yes	38.94 %	48.39 %	43.20 %	0.1731
No	61.06 %	51.61 %	56.80 %	
Psychosocial factors and impact of pain				
FABQ Activity [mean (SD)]	16.48 (6.13)	14.29 (7.49)	15.49 (6.85)	0.0517
FABQ Work [mean (SD)]	27.96 (11.88)	24.56 (10.64)	26.41 (11.42)	0.0698
QUEBEC [mean (SD)]	37.35 (15.67)	31.97 (19.53)	34.91 (17.67)	0.0615
HAD Anxiety Scale [mean (SD)]	10.08 (4.26)	9.01 (4.48)	9.60 (4.38)	0.1266
HAD Depression Scale [mean (SD)]	7.62 (5.33)	6.07 (3.82)	6.92 (4.75)	*0.0345*
DALLAS Anxiety/Depression [mean (SD)]	42.47 (26.77)	33.92 (26.13)	38.61 (26.74)	*0.0412*
DALLAS Work and Leisure [mean (SD)]	58.28 (20.42)	51.68 (24.85)	55.30 (22.70)	0.0637
DALLAS Daily Activities [mean (SD)]	60.54 (16.62)	52.58 (21.38)	56.95 (19.27)	*0.0098*
DALLAS Sociability [mean (SD)]	33.89 (26.47)	24.26 (20.25)	29.54 (24.28)	*0.0110*
Interventions				
Workplace interventions				
Yes	49.5 % (58)	49.5 %(49)	55,36 %	
No	50.5 % (60)	50.5 % (50)	44,64 %	
Physiotherapy				
None	6.06 %	5.13 %	5.65 %	*0.0125*
Trained Physiotherapist	54.55 %	33.33 %	45.20 %	
Not Trained Physiotherapist	39.39 %	61.54 %	49.15 %	
Physiotherapy [number of sessions**,** mean (SD)]	24.30 (31.94)	20.55 (12.11)	22.68 (25.36)	0.8205

Comparison of means by Student’ test or Wilcoxon’ test. Comparison of % by *X* ^2^ test or fisher’ test

Professional Data: Job satisfaction: 78 % of patients reported job satisfaction with no significant difference between the two groups, but the number of days’ sickness leave absence before inclusion was significantly higher in the non-HCW group (mean of 174.1 days versus 93.5 days; *p* = 0.0002).

Data relating to intervention: intervention in the workplace by the OP or guidance to change the job was involved in 49.5 % of cases in both groups. However the type of interventions were different for the two groups. In the HCW group, most interventions involved reclassification to “soft” jobs such as reception, admissions for nursing aides, technicians, or clinical research assistants for nurses. In the control group, there were most adjustments in the working conditions, such as modification of schedules, temporary part-time work, temporary drop loads to be handled, or ergonomic design of seats for drivers. The type of rehabilitation program did not differ between the two groups in terms of numbers of interventions, but differed according to the group in terms of orientation: carers more often consulted network physiotherapists, while employees in the private sector consulted their usual physiotherapist more frequently (*p* = 0.0125).

- o Clinical and morphological data: weight and size were lower in the HCW group but the mean BMI was the same.
- o Concerning pain before inclusion in the program: The pain level was lower in the HCW group (3.33 versus 4.53). Duration of pain was not significantly different between the two groups (HCW/92 days - non HCW/72 days, *p* = 0.0001). As regards the history of lumbago, radiculopathy, or the presence of comorbidity, including of psychiatric origin, there was no significant difference between the two groups.
- o Psychosocial variables and impact of pain assessed by psychometric tests: scores for depression and anxiety (HAD) were slightly higher in the non-HCW group (depression = 7.62 versus 6.07; *p* = 0.034) but the mean scores remained within the thresholds of risk (10/20). Scores for avoidance of fear (FABQ) in the face of pain were similar in both groups at baseline. The impact of pain on the level of anxiety/depression, on daily activities and sociability (DALLAS) were significantly higher in the non-HCW group (daily activity = 60.5 versus 52.6; *p* = 0.009) (Table 1).

When analyzing the overall effectiveness of the program, effectiveness had been maintained at two years in terms of functional criteria (fingertip-floor distance), psychological factors including reduced apprehension and avoidance in relation to pain, decreased impact of pain on professional activities, daily activities and recreation (Table 2).

**Table 2:** Analysis of two year effectiveness of the rehabilitation program (T1 = start of the study, T4 = end of the study); only variables with a statistically significant effect (*p* < 0.05) after two years are reported in the table

Variables	N	T1	T4	T4 - T1	*p*-value
**◊** Clinical factors					
Fingertip-floor distance [cm, mean (SD)]	64	21.96 (18,12)	13.88 (13,36)	- 8.09 (17,40)	<0.0001
**◊** Psychosocial factors					
FABQ activity [mean (SD)]	54	15.85 (7,11)	13.35 (7,20)	−2.50 (7,60)	0.0191
FABQ work [mean (SD)]	54	27.24 (12,46)	23.57 (12,02)	−3.67 (11,46)	0.0225
QUEBEC [mean (SD)]	55	38.05 (20,24)	31.56 (21,44)	−6.49 (18,03)	0.0100
DALLAS work and leisure [mean (SD)]	60	57.00 (26,62)	48.95 (28,41)	−8.05 (26,74)	0.0231
DALLAS daily activities [mean (SD)]	60	58.13 (28,48)	48.98 (25,99)	−9.15 (22,62)	0.0027

- o The overall “staying at work” rate was 77 % at two years (Table 3).

**Table 3:** Potential predictive factors of return to work (RTW) at baseline (T1)

Predictive factors at T1		RTW = No	RTW = Yes	Total RTW	*p*-value
		*N* = 27	*N* = 92	*N* = 119	
Sociodemographic factors					
Age	Mean (SD)	42.07 (8.69)	39.83 (9.65)	40.42 (10.19)	0.3177
Sex (%)	Female	37.4	58.70	53.78	0.0472
	Male	62.9	41.3	46.22	
Conjugal relationship (%)	Yes	42.31	44.44	43.97	0.8467
	No	57.69	55.56	56.03	
Pain level					
Duration of pain (months)	Mean (SD)	99.48 (87.58)	85.07 (88.93)	84.34 (88.47)	0.2793
VAS (Visual Analogue Scale 1–10)	Mean (SD)	4.34 (2.29)	3.74 (2.23)	3.87 (2.25)	0.2976
Repetitive Lumbago history	Yes	66.67	57.61	59.66	0.3989
	No	33.33	42.39	40.34	
Recurrent Lombosciatica	Yes	48.15	45.65	46.22	0.8191
	No	51.85	54.35	53.78	
Comorbidity	0	34.62	58.43	53.04	0.0913
(0 = none; 1 = cardiovascular, others…; 2 = psychiatric)	1	42.31	29.21	32.17	
	2	23.08	13.36	14.78	
Tobacco (packs.years)	Mean (SD)	5.90 (11.50)	3.06 (6.39)	3.65 (7.74)	0.3137
Clinical examination data					
Weight (Kg)	Mean (SD)	72.37 (16.30)	68.61 (14.39)	69.47 (14.86)	0.2868
Height (cm)	Mean (SD)	170.58 (9.40)	169.33 (9.18)	169.61 (9.20)	0.5218
Body Mass Index (kg/m2)	Mean (SD)	24.96 (5.85)	23.81 (4.20)	24.07 (4.62)	0.4924
Fingertip-floor distance (cm)	Mean (SD)	26.65 (15.63)	17.03 (14.23)	19.15 (15.02)	0.0053
Schober index (10-cm Schober test)	Mean (SD)	2.94 (1.08)	3.22 (1.07)	3.16 (1.07)	0.4908
Occupational factors					
Absenteeism before inclusion (days)	Mean (SD)	196 (177)	111 (131)	129 (145)	0.0151
Work Satisfaction (%)	Yes	60.00	80.50	76.50	0.076
	No	40.00	19.50	23.50	
Occupation (%)	HCWs	11.11	56.52	46.22	<0.0001
	No Hcws	88.89	43.48	53.78	
Psychosocial factors and impact of the pain					
FABQ activity	Mean (SD)	16.67 (6.04)	14.56 (7.54)	14.96 (7.29)	0.1466
FABQ work	Mean (SD)	30.50 (9.47)	25.97 (12.44)	26.78 (12.04)	0.2795
QUEBEC scale	Mean (SD)	46.73 (17.14)	33.84 (19.40)	36.35 (19.56)	0.0272
HAD scale anxiety	Mean (SD)	10.25 (4.60)	9.00 (4.33)	9.24 (4.38)	0.3724
HAD scale Depression	Mean (SD)	7.69 (4.06)	6.33 (4.09)	6.59 (4.10)	0.1901
DALLAS Anxiety/Depression	Mean (SD)	57.19 (27.63)	37.64 (27.31)	41.32 (28.26)	0.0156
DALLAS Work and leisure	Mean (SD)	68.13 (18.98)	54.71 (24.59)	57.24 (24.12)	0.0610
DALLAS Daily activities	Mean (SD)	66.47 (14.98)	55.33 (20.89)	57.42 (20.31)	0.0644
DALLAS Sociability	Mean (SD)	44.84 (27.33)	29.17 (23.66)	32.12 (24.99)	0.0467
Intervention factors

The favorable prognostic factors at baseline for staying at work were, in our study, to be a carer, to have fewer than 112 days’ sick leave, a small initial fingertip-floor distance, and low scores for the impact of anxiety, depression and daily discomfort. Satisfaction at work was a good predictor of staying at work after 2 years. (Tables 3 and 4).

**Table 4:** Potential predictive factors for Return to Work: Healthcare Workers versus Non-Healthcare Workers

Predictive factors for RTW		« Healthcare Worker » group				« Non-Healthare Worker » group			
		RTW = No RTW = Yes Total *N* = 3 *N* = 52 *N* = 55			*p*-value	RTW = No RTW = Yes Total *p*-value *N* = 24 *N* = 40 *N* = 64			
Sociodemographic factors									
Age	Mean (SD)	43.67 (6.51)	40.06 (10.37)	40.25 (10.19)	0.5927	41.88(9.02)	39.78(8.48)	40.56(8.68)	0.3452
Sex	Female	3 (100.0 %)	45 (86.54 %)	48 (87.27 %)	1.0000	7 (29.17 %)	9 (22.50 %)	16 (25.00 %)	0.5510
	Male	0 (0.00 %)	7 (13.46 %)	7 (12.73 %)		17 (70.83 %)	31 (77.50 %)	48 (75.00 %)	
Conjugal relationship	Yes	1 (33.33 %)	23 (46.00 %)	24 (45.28 %)	1.0000	10 (43.48 %)	17 (42.50 %)	27 (42.86 %)	0.9398
	No	2 (66.67 %)	27 (54.00 %)	29 (54.72 %)		13 (56.52 %)	23 (57.50 %)	36 (57.14 %)	
Pain level									
Duration of pain (months)	Min-Max	[12.00,’72.00]	[2.00,384.00]	[2.00,384.00]	0.5547	[5.00,336.00]	[5.00,324.00]	[5.00,336.00]	0.2882
	Mean (SD)	35.33 (32.15)	83.38(90.69)	80.76(89.04)		107.50(89.31)	87.25(87.69)	94.84(88.15)	
	Median	22.00	36.00	36.00		102.00	60.00	66.00	
VAS Visual Analogue Scale TO	Min-Max	[2.00,3.00]	[0.00,9.00]	[0.00,9.00]	0.7343	[0.50,8.00]	[0.00,8.00]	[0.00,8.00]	0.9824
	Mean (SD)	2.50 (0.71)	3.14 (2.26)	3.11 (2.22)		4.51 (2.32)	4.48 (1.99)	4.49 (2.09)	
	Median value	2.50	3.00	3.00		4.55	5.00	5.00	
Repetitive Lumbago history	Yes	3 (100.0 %)	30 (57.69 %)	33 (60.00 %)	0.2667	15 (62.50 %)	23 (57.50 %)	38 (59.38 %)	0.6934
	No	0 (0.00 %)	22 (42.31 %)	22 (40.00 %)		9 (37.50 %)	17 (42.50 %)	26 (40.63 %)	
Recurrent Lumbosciatica	Yes	2 (66.67 %)	27 (51.92 %)	29 (52.73 %)	1.0000	11 (45.83 %)	15 (37.50 %)	26 (40.63 %)	0.5111
	No	1 (33.33 %)	25 (48.08 %)	26 (47.27 %)		13 (54.17 %)	25 (62.50 %)	38 (59.38 %)	
Comorbidity (0 = none; 1 = cardiovascular,	0	1 (33.33 %)	33 (66.00 %)	34 (64.15 %)	0.3853	8 (34.78 %)	19 (48.72 %)	27 (43.55 %)	0.5871
	1	2 (66.67 %)	13 (26.00 %)	15 (28.30 %)		9 (39.13 %)	13 (33.33 %)	22 (35.48 %)	
others…;2 = psychiatric)	2	0 (0.00 %)	4 (8.00 %)	4 (7.55 %)		6 (26.09 %)	7 (17.95 %)	13 (20.97 %)	
Tobacco (packs.years)	Min-Max Mean (SD)	[0.00,0.00]	[0.00,15.00]	[0.00,15.00]	0.5277	[0.00,45.00]	[0.00,30.00]	[0.00,45.00]	0.7779
		0.00 (0)	1.64	1.57		6.53	5.03	5.57	
Clinical examination data									
Weight (Kg)	Min-Max Mean (SD)	[62.00,70.00]	[42.00,90.00]	[42.00,90.00]	0.5799	[43.00,118.00]	[43.00,117.00]	[43.00,118.00]	0.7507
		66.17 (4.01)	64.55	64.64		73.15	73.90	73.62	
Height (cm)	Min-Max Mean (SD)	[160.00,160.00]	[150.00,187.00]	[150.00,187.00]	0.1520	[153.00,190.00]	[150.00,190.00]	[150.00,190.00]	0.4942
		160.00 (0)	166.58 (7.60)	166.33 (7.56)		171.46 (9.25)	172.85 (9.90)	172.32 (9.61)	
BMI	Mean	25.78 (2.21)	23.25 (4.40)	23.35 (4.35)	0.2491	24.89 (6.07)	24.53 (3.87)	24.67 (4.78)	0.7247
Fingertip-floor distance (cm)	Min-Max	[10.00,37.00]	[−5.00,90.00]	[−5.00,90.00]	0.6308	[0.00,57.00]	[0.00,45.00]	[0.00,57.00]	0.0289
	Mean (SD)	19.67 (15.04)	16.47 (15.96)	16.65 (15.79)		27.57 (15.79)	17.76 (11.76)	21.34 (14.07)	
	Median	12.00	16.50	16.00		25.00	19.00	22.00	
	value								
Schober index (cm)	Min-Max	[2.00,4.00]	[1.00,7.00]	[1.00,7.00]		[0.00,5.00]	[1.50,5.00]	[0.00,5.00]	0.5258
	Mean (SD)	3.00 (1.00)	3.23 (1.16)	3.22 (1.14)		2.93 (1.11)	3.22 (0.94)	3.10 (1.01)	
	Median value	3.00	3.00	3.00		3.00	3.00	3.00	
Socio-occupational factors									
Absenteeism	Min-Max	[0.00,365.00]	[0.00,570.00]	[0.00,570.00]	0.7976	[0.00,720.00]	[0.00,540.00]	[0.00,720.00]	0.1189
Before inclusion (days)	Mean (SD)	142 (196)	91 (120)	94 (123)		204 (178)	134 (142)	161 (158)	
	Median value	60.00	47.00	53.50		140.00	83.50	110.00	
Job Satisfaction	Yes	1 (50.00 %)	36 (78.26 %)	37 (77.08 %)	0.4096	11 (61.11 %)	30 (83.33 %)	41 (75.93 %)	0.0961
	NO	1 (50.00 %)	10 (21.74 %)	11 (22.92 %)		7 (38.89 %)	6 (16.67 %)	13 (24.07 %)	
Psychosocial factors and impact of pain									
FABQ activity	Min-Max	[14.00,’24.00]	[0.00,42.00]	[0.00,42.00]	0.2288	[3.00,24.00]	[0.00,24.00]	[0.00,24.00]	0.3785
	Mean (SD)	18.67 5.03)	14.34 (8.56)	14.68 (8.37)		16.17 (6.37)	14.83 (6.22)	15.22 (6.21)	
	Median value	18.00	13.00	13.50		18.00	15.00	16.00	
FABQ work	Min-Max	[30.00,40.00]	[0.00,42.00]	[0.00,42.00]	0.1124	[14.00,42.00]	[2.00,42.00]	[2.00,42.00]	0.8801
	Mean (SD)	34.33 (5.13)	23.31 (12.36)	24.18 (12.28)		29.45 (10.28)	29.17 (11.98)	29.25 (11.41)	
	Median value	33.00	23.00	25.50		32.00	33.00	33.00	
QUEBEC scale	Min-Max	[34.00,71.00]	[5.00,72.00]	[5.00,72.00]	0.2717	[16.00,80.00]	[3.00,65.00]	[3.00,80.00]	0.0855
	Mean (SD)	46.67 (21.08)	32.58 (21.35)	33.75 (21.40)		46.7 (17.12)	35.28 (17.16)	38.63(17.74)	
	Median value	35.00	28.00	33.50		48.50	33.00	38.00	
HAD scale anxiety	Min-Max	[4.00,15.00]	[1.00,19.00]	[1.00,19.00]	0.2726	[3.00,18.00]	[3.00,17.00]	[3.00,18.00]	0.7932
	Mean (SD)	11.00 (6.08)	7.86 (4.31)	8.11 (4.45)		10.08 (4.48)	10.25 (4.05)	10.20 (4.13)	
	Median value	14.00	8.00	8.00		10.00	10.00	10.00	
HAD scale Depression	Min-Max	[5.00,10.00]	[0.00,14.00]	[0.00,14.00]	0.2182	[1.00,13.00]	[1.00,18.00]	[1.00,18.00]	0.6710
	Mean (SD)	8.00(2.65)	5.54(4.00)	5.74 (3.94)		7.62 (4.41)	7.19 (4.08)	7.31 (4.13)	
	Median value	9.00	4.00	5.00		9.00	7.00	7.00	
DALLAS Anxiety/ Depression	Min-Max	[50.00,80.00]	[0.00,85.00]	[0.00,85.00]	0.0785	[0.00,100.00]	[0.00,90.00]	[0.00,100.00]	0.1862
	Mean (SD)	60.00 (17.32)	31.28 (26.18)	33.44 (26.59)		56.54 (30.03)	45.00 (27.11)	48.33 (28.14)	
	Median value	50.00	25.00	30.00		65.00	48.75	55.00	
DALLAS Work and leisure	Min-Max	[35.00,85.00]	[2.50,90.00]	[2.50,90.00]	0.6099	[40.00,95.00]	[5.00,90.00]	[5.00,95.00]	0.1294
	Mean (SD)	61.67 (25.17)	51.88 (26.72)	52.61 (26.43)		69.62 (18.22)	57.98 (21.82)	61.34 (21.32)	
	Median value	65.00	57.50	60.00		70.00	60.00	65.00	
DALLAS	Min-Max	[42.00,90.00]	[0.00,81.00]	[0.00,90.00]	0.3219	[45.00,87.00]	[24.00,93.00]	[24.00,93.00]	0.3713
Daily activities	Mean (SD)	66.00 (24.00)	49.86 (21.61)	51.08 (21.89)		66.58 (13.58)	61.64 (18.38)	63.07 (17.13)	
	Median value	66.00	54.00	55.50		69.00	64.50	66.00	
DALLAS	Min-Max	[35.00,45.00]	[0.00,75.00]	[0.00,75.00]	0.1345	[10.00,85.00]	[0.00,90.00]	[0.00,90.00]	0.5172
Sociability	Mean (SD)	40.00 (5.00)	22.70 (20.80)	24.00 (20.54)		45.96 (30.37)	36.64 (24.86)	39.33 (26.56)	
	Median value	40.00	20.00	20.00		50.00	35.00	35.00	
Intervention factors									
Ergonomic and OP intervention	Yes	0 (0.00 %)	24 (47.06 %)	24 (45.28 %)	0.4949	12 (54.55 %)	26 (70.27 %)	38 (64.41 %)	0.2225
	No	2 (100.0 %)	27 (52.94 %)	29 (54.72 %)		10 (45.45 %)	11 (29.73 %)	21 (35.59 %)	
Physiotherapy 0 = none 1 = classic	0	1 (33.33 %)	1 (2.04 %)	2 (3.85 %)	0.0177	2 (8.70 %)	2 (5.56 %)	4 (6.78 %)	0.7026
	1	2 (66.67 %)	17 (34.69 %)	19 (36.54 %)		13 (56.52 %)	18 (50.00 %)	31 (52.54 %)	
2 = trained physiotherapist	2	0 (0.00 %)	31 (63.27 %)	31 (59.62 %)		8 (34.78 %)	16 (44.44 %)	24 (40.68 %)	
Physiotherapy	Yes	1 (33.33 %)	1 (2.00 %)	2 (3.77 %)	0.1110	2 (8.33 %)	2 (5.41 %)	4 (6.56 %)	0.6427
	No	2 (66.67 %)	49 (98.00 %)	51 (96.23 %)		22 (91.67 %)	35 (94.59 %)	57 (93.44 %)	
	Min-Max	[0.00,’20.00]	[0.00,100.00]	[0.00,100.00]	0.5231	[0.00,300.00]	[0.00,60.00]	[0.00,300.00]	0.2150
Physiotherapy (number of training sessions)	Mean (SD)	13.33 (11.55)	20.70 (13.53)	20.28 (13.44)		41.13 (62.31)	20.32 (12.30)	28.51 (41.04)	

Univariate analysis showed some positive prognostic factors for those included in the study for returning to or staying at work : being a carer (OR 0.1; 95 % CI [0.03–0.34]), a low fingertip-floor distance (OR 0.96; 95 %o CI [0.93; 0.99]), a low anxiety/depression score (OR 0.97; 95 %o CI [0.95; 1.00]), the functional impact of the pain (OR 0.96; 95 % CI [0.93–1.00]), and the impact of the pain on social relationships (OR 0.98; 95 % CI [0.95–1.00]) (Table 5).

**Table 5:** Odds ratios (OR) and confidence interval for keeping job after 2 years

Pronostic factors	N	OR	IC 95 %	*p*-value
Carer: no vs yes	119	0.10	[0.03; 0.34]	*0.0003*
Fingertip-floor distance	118	0.96	[0.93; 0.99]	*0.0079*
DALLAS Anxiety/Depression	85	0.97	[0.95; 1.00]	*0.0164*
Sick leave	108	1.00	[0.99; 1.00]	*0.0190*
QUEBEC	77	0.96	[0.93; 1.00]	*0.0267*
DALLAS Social comportment	85	0.98	[0.95; 1.00]	*0.0282*
Gender : female vs male	119	2.42	[1.00; 5.85]	0.0506
DALLAS work and leisure	85	0.97	[0.94; 1.00]	0.0523
DALLAS daily activities	85	0.97	[0.94; 1.00]	0.0523
Work satisfaction: no vs yes	102	0.36	[0.13; 1.04]	0.0585
Physiotherapy (0 = none; 1 = liberal; 2 = trained physiotherapy) 0 vs 2	111	0.17	[0.03; 1.00]	0.0621
Physiotherapy (0 = none; 1 = liberal; 2 = trained physiotherapy) 1 vs 2	111	0.40	[0.15; 1.04]	.
Comorbidity (0 = none; 1 = other; 2 = psy) 0 vs 2	115	3.15	[0.93; 10.68]	0.1006
Comorbidity (0 = none; 1 = other; 2 = psy) 1 vs 2	115	1.29	[0.38; 4.36]	.
Physiotherapy (n sessions): 0 vs >0	114	0.29	[0.05; 1.51]	0.1399
FABQ work	78	0.97	[0.91; 1.02]	0.2060
HAD Depression	83	0.92	[0.81; 1.05]	0.2351
Pain intensity	113	0.89	[0.72; 1.09]	0.2465
Weight	119	0.98	[0.96; 1.01]	0.2495
Schober index	106	1.30	[0.83; 2.02]	0.2507
Body mass index	115	0.95	[0.87; 1.04]	0.2731
Age	119	0.97	[0.93; 1.02]	0.2972
HAD scale anxiety	83	0.94	[0.82; 1.06]	0.3055
FABQ activity	79	0.96	[0.89; 1.04]	0.3160
Cigarette smoking : 0 vs >0	102	1.65	[0.60; 4.52]	0.3296
Lumbago history : no vs yes	119	1.47	[0.60; 3.62]	0.4005
Pain duration	119	1.00	[0.99; 1.00]	0.4562
Height	115	0.99	[0.94; 1.03]	0.5410
Sciatalgia history : no vs yes	119	1.11	[0.47; 2.61]	0.8191
Maried : no vs yes	116	0.92	[0.38; 2.21]	0.8467

Level of significance based on logistic regression analysis. *p* < 0.05 in italics.

Intervention by the physiotherapist seemed to show its effectiveness particularly when designed to support the chronic LBP with longer sessions (one hour) focusing on stretching and not on massage or focusing on self-rehabilitation at home; the effect was maintained over time. Intervention at the workplace did not seem to affect staying at work at this stage of the analysis. After multivariate analysis, only occupation remained a good prognosis factor for job retention at two years follow up (Table 6).

**Table 6:** Potential predictive factors of RTW: multivariate analysis, only variables with *p* < 0.15 were selected

Potential predictive factors	N	OR	IC	*p*-value
Absenteeism	71	1.00	[0.99; 1.01]	0.6562
Comorbidity : 0 vs 2		1.95	[0.12; 32.08]	0.2061
Comorbidity : 1 vs 2		0.23	[0.02; 3.38]	
Physiotherapy		0.95	[0.87; 1.04]	0.2629
Satisfaction at work: no vs yes		0.13	[0.01; 1.52]	0.1045
HCW : no vs yes		0.02	[0.00; 0.62]	0.0249
DALLAS anxiety/depression		0.96	[0.90; 1.02]	0.1577
DALLAS work and leisure		0.99	[0.92; 1.06]	0.7587
DALLAS daily activities		1.04	[0.94; 1.14]	0.4413
Fingertip-floor distance		0.98	[0.89; 1.09]	0.7639
FABQ work		1.07	[0.95; 1.21]	0.2340
Quebec		0.97	[0.89; 1.06]	0.5185
Sex: female vs male		0.08	[0.00; 1.45]	0.0878
Sociability		1.00	[0.95; 1.06]	0.9445

## Discussion

To our knowledge, this is the first study that analyses RTW prognosis factors of workers with CLBP at 2 years’ follow-up. The studies proposed in the scientific literature are based first on RTW rates and second on the method of CLBP management (individual = AIT or multidisciplinary = RFP). In our study, we found 77.3 % of RTW after 2 years of follow up. To date, we found in the literature only 5 weeks to 1 year of follow-up studies with very different return-to-work rates : 86 % at 5 weeks follow-up and 60 % at 3 months (12) [12], 70 % at one year [13], and 72 % completely recovered at 1 year [14]. One study compared FRP versus AIT at 6 months and demonstrated 69 % RTW with FRP versus 66.7 % RTW with AIT [15]; another showed 22 % RTW with only a brief vocationally oriented intervention, but there was no statistical difference from the control group [16]. Our study, with two years of follow up, highlighted predictive factors for staying at work before inclusion in the rehabilitation program, such as being a healthcare worker, a low rate of absenteeism, and care by a well-trained physiotherapist (physiotherapist of the Regional Network). Regarding the impact of physiotherapy, we compared “standard care” by the physiotherapist (private financing), with treatment by a physiotherapist of the regional network which is more active, with stretching, muscular strengthening, and for longer sessions (private rehabilitation center with additional public financing). Our results showed that intervention by a trained physiotherapist to take care of CLBP might have very good results as regards preservation of the employment (univariate analysis). That would show the specificity of this type of rehabilitation and the need to train more physiotherapists to understand reconditioning during strain the education of patients through lifestyle advice and advice on good movements and postures for carrying loads or repetitive movements, to adapt exercises to each patient, including enabling auto-rehabilitation at home. In our study, HCWs had less often trained physiotherapists (Table 1), however they returned more often to work (Table 3). This may be explained by the fact that we have included in the study HCWs working in large public-sector organizations.

Similarly, 17 out of 19 HCWs with classic physiotherapy returned to work, 31 out of 31 with trained physiotherapists returned to work and the *p*-value for the HCW is *p* = 0.0177 (Table 4) : this is the test for trend including the no therapy group; this *p*-value is based on two successful observations only and therefore is not robust.

In addition, our study highlights predictors of job retention with a two-year follow-up, with a positive effect of the occupational factor (56.52 % versus 43.48 %; *p* < 0.0001). That was confirmed by multivariate analysis. This better level of RTW for HCW can be potentially explained by a better method of reintegrating public healthcare employees in France. Rehabilitation shows an overall positive effect on physical parameters but both rehabilitation programme and intervention in the workplace does not seem to predict job retention. The studies published so far about the healthcare sector show a strong link between CLBP and biomechanical risk factors, CLBP and psychosocial risk factors as evidenced by numerous cohort studies [6, 17–22] and recent case–control studies [23–27]. There is no study in the literature to date showing the effectiveness of dual management involving both functional rehabilitation and action at the workplace. Nonetheless, further research is needed on possible interactions between the effect of intervention at the workplace and the effect of the physiotherapy factor.

On the level of absenteeism, people who have been on long-term sickness leave seem to have greater difficulty in returning to work. This variable was highlighted in some studies but only after 6 or 12 months [13]. Concerning the clinical criteria, a low fingertip-floor distance seems to be a factor for good prognosis; the initial severity of the pain and RTW are not linked as evidenced by other authors who highlighted chronicity factors such as the intensity of the initial pain, apprehension, psychosocial factors and the intensity of the work [17, 19]. Physiotherapeutic care with education of the patients could decrease this “phobia” of movement and apprehension of pain that probably play a role in the delayed resumption of activities. We also highlighted a significant improvement in the scores estimating the perception of the pain and the psychosocial data, the decrease in apprehension of the pain between T1 and T4 (Table 2). Initial pain is thus a variable important to consider, but we should not neglect the psychosocial aspects, being able to be at the origin of a resumption of work pushed away that we were able to highlight in our study through the analysis of the QUEBEC and DALLAS questionnaires.

In our study, interventions in the work environment did not seem to predict significantly job retention. However only 50 % of the employees in the both groups (HCW and non-HCW) had one intervention at their workplace after 2 years. These were most often implemented by the OP of the company, the supervisor, or on the employee’s initiative. The difference between the HCW group and the non-HCW group could be due to the various interventions and the proposals made in every group, as for example: in the hospital sector, professional reassignment usually takes place in admission consultation, outpatient services, telephone reception, post office consultation; while in the industrial or commercial private sector, it especially involves ergonomic arrangements, set-up of workstations with purchase of an adapted seat, change of desk, or ample room to move, ergonomic arrangement of the controls or modification of the traveled distance for the commercial workers. In France, another by no means obvious element to consider, is the possibility of a therapeutic or part-time/half-time resumption of work in the General Healthcare Insurance System for the private sector; while for public workers part-time or half-time resumption of work is possible only after at least 6 months of sick leave, which is most probably a brake on an early return to work. The best way to intervene would thus be to identify patients with a high risk of chronic pain and introduce preventive measures to reduce the risk of delayed healing. Interventions in the work environment and physiotherapy seem to be obvious ways to achieve this, thereby enabling a resumption of activities in the best possible conditions. This is what we tried to set up in this study over 2 years, with 6 months, 1 and 2 year follow-up consultations. The repetition of those consultations allowed us to adjust the care before the end of the program, for example by proposing new physiotherapy sessions and/or adjustments to work stations or schedules. They also made it possible to safeguard a professional activity that would not have been possible with a more intensive program requiring work schedule arrangements or hospitalization as in the most intensive programmes. To date, we find no study in the literature showing the efficiency of double coverage involving both simultaneous intervention by a trained physiotherapist and action at the workplace. However, our study needs to follow up its investigations by quantifying more exactly the types of job design and the modalities of resumption of work in the private and public sector.

### Our study has some limitations

An insufficient number of trained therapists, the low number of subjects (*N* = 120) and a large number of variables, the number of lost participants (67 % at two years, reduced to 22 % thanks to the program of phone call reminders and emails), the absence of randomization, so it is not really a RCT. The two groups are non-comparable in terms of sex, professional sector (public-private), but comparable in terms of number of interventions in the workplace and number of sessions of physiotherapy. At this stage, our multivariate analysis should be approached with cautions.

Our rehabilitation programme with trained physiotherapists, and interventions in the workplace did not show statistically significant effectiveness probably because of lack of power of the study; indeed, our cohort should be developped.

Interventions in the workplace are not of the same type in hospitals and in the “control area”, especially in the industrial sector: in the first case, the employees especially benefited from professional outplacement to suitable posts such “advice”, phone reception, courier in the second case, it was mainly ergonomic improvements with purchase of ergonomic seats, changes of work tables, modifying career path for businessmen. Our study therefore requires further investigation involving quantifying more precisely the types of work adjustments by sector. Another area to investigate would be the return to work modalities, which vary between the public and the private sector in France. In the private sector, employees are affiliated to the General Regime of Social Security and physicians may ask for any person who has stopped work a “therapeutic half-time” or a “therapeutic part-time” return to work. In the public sector, French legislation prescribes a period of six months off work before allowing a half-time or part-time return to work, which is probably an obstacle to an early return to work. Overall, these considerations deserve to be supported by a further study.
